# Accuracy of CAD/CAM planned mandibular reconstruction with scapula free flaps in reconstructive head and neck surgery – A single center study

**DOI:** 10.1007/s10006-026-01505-z

**Published:** 2026-02-04

**Authors:** Jonas Wüster, Max Brandenburg, Tim Lukas Elter, Norbert Neckel, Jakob Fenske, Christian Doll, Carsten Rendenbach, Max Heiland, Claudius Steffen, Kilian Kreutzer, Steffen Koerdt

**Affiliations:** 1 Department of Oral and Maxillofacial Surgery, Faculty of Medicine, University Medical Center Freiburg, Albert Ludwig University of Freiburg, Baden- Württemberg, Germany; 2 Department of Oral and Maxillofacial Surgery, Charité – Universitätsmedizin Berlin, corporate member of Freie Universität Berlin and Humboldt-Universität zu Berlin, Berlin, Germany

**Keywords:** Scapula free flap, CAD/CAM, Mandibular reconstruction, Patient-specific implant, Free flaps, Accuracy, Microvascular transplant, Free bone transplant

## Abstract

**Purpose:**

For the first time, this study explores the transfer precision of computer aided design and computer aided manufacturing (CAD/CAM) in mandibular reconstruction with scapula free flaps (SFF).

**Methods:**

Patients undergoing mandibular reconstruction with SFF and patient-specific 3D-printed titanium plates were retrospectively analyzed. To assess planning accuracy, pre- and postoperative datasets were overlaid using 3D Slicer software. Specific measurements included the fit at the mandibular resection site, intersegmental gap width between scapula and mandible, scapular segment positioning, and condylar alignment. Osseous union was evaluated via postoperative computed tomography (CT) or cone beam CT, and plate-related complications were recorded clinically.

**Results:**

In total 15 patients (mean age 65.7 ± 10.0 years) were included. Postoperative scapular segment positioning showed deviations in the posterior region (basal: 6.78 ± 3.34 mm; crestal: 7.61 ± 3.40 mm) and anterior region (basal: 3.54 ± 1.52 mm; crestal: 4.49 ± 2.51 mm). Mean deviation in segmental gap width at the anterior and posterior sites ranged from 0.55 to 1.72 mm and was statistically significant at all four measurements. Mean contralateral condyle position differed significantly from the initial position (8.56 ± 4.31 mm). Intraoral plate exposure occurred in three patients without adjuvant radiotherapy; no extraoral exposure, fractures, or loosening were observed. Non-union between the mandible and SFF was found in three patients.

**Conclusion:**

As with other CAD/CAM-assisted mandibular reconstructions, SFF exhibit discrepancies between virtual planning and the postoperative outcome. The surrounding soft tissue bulk of the scapula can hinder the precise fit of cutting guides, potentially compromising reconstruction accuracy and contributing to increased deviations. Despite these challenges, CAD/CAM-assisted SFF demonstrates acceptable surgical predictability.

## Introduction

 In the treatment of benign and malignant conditions located near the mandible, particularly oral cancer, segmental mandibulectomy is often performed [1]. The resulting segmental defects normally exceed the intrinsic bone healing capacity [2], wherefore the need for osseous reconstruction of the mandible arises. Regarding osseous reconstruction, autologous bone is still considered to be the “gold standard” [3, 4]. Consequently, osseous free flaps with microsurgical anastomosis are normally used for primary or secondary reconstruction [5, 6]. For reconstruction of the mandible, especially after oncological resection, the free fibula flap (FFF) is seen as the “gold standard”, due to its sufficient quantity of bone, the possibility of ideal contouring, vascular supply and a long pedicle [7–9]. Nevertheless, there are also reasons for using alternatives, e.g. the scapular free flap (SFF). Dowthwaite et al., consider free flaps of the subscapular system as excellent options for elderly patients, those with significant comorbidities, such as peripheral vascular disease, and mandibular defects associated with complex soft-tissue requirements [10]. It has also been demonstrated, that the scapular tip free flap (STFF) is associated with low morbidity, and early ambulation time [11]. Moreover, the SFF offers the possibility to be used as a chimeric flap, enabling the reconstruction of mandibular defects associated with complex soft-tissue requirements [10, 12]. When comparing FFF and SFF in head and neck reconstruction, Liu et al. reported longer surgery times for SFF, but less early morbidity [13]. Dimovska et al. reached a similar conclusion with superior donor site outcomes for SFF when compared to FFF [14]. It was additionally shown, that the concave shape of the scapula allowed for fewer osteotomies in curved reconstruction, which was particularly noticeable in lateral mandibular defects [14]. The authors concluded that the SFF may offer advantages in osseous head and neck reconstructions by facilitating “non-osteotomized and chimeric reconstructions without compromising surgical outcomes or quality of life” [14].

Lately, the introduction of computer-aided-design and computer-aided-manufacturing (CAD/CAM) has been incorporated into the surgical workflow of reconstructive surgery. Particularly osseous reconstruction of the mandible with free flaps has been revolutionized due to CAD/CAM in combination with initially milled and then selective laser-melted patient-specific plates [15, 16]. Prior to the surgery, high resolution computed tomography (CT)-scans are generally evaluated, and virtual planning is carried out [17]. This virtual plan is later transferred to the operation theater via surgical guides and patient specific implants (PSI) [18, 19]. Since bending of PSIs is not required, a higher stability of the osteosynthesis is guaranteed [20, 21]. Besides that, recent studies proofed that CAD/CAM techniques enable to decrease the total operation time, the ischemic time of the transplant and subsequent the length of hospitalization [22–25].

When comparing conventional and CAD/CAM methods, previous studies have pointed out that CAD/CAM enables significant improvements in accuracy [26] in mandibular reconstruction using FFF [27–29], or the deep circumflex iliac artery (DCIA) flap [30]. Despite the advances, the final postoperative results often contain deviances of several millimeters [29, 31]. Since the accuracy of the virtual surgical planning (VSP) transferred to the actual result is not only of importance for the patient’s aesthetic outcome, it also directly affects the functional outcome. Moreover, correct positioning of the bony segments is essential for later dental implant placement and subsequent successful prosthetic rehabilitation as well as prevention of osseous non-unions. Therefore, this study aims to investigate the accuracy of SFF in mandibular reconstruction, with special emphasize on the postoperative positioning of the scapular segment(s), the segmental gap width and condylar positioning.

## Materials and methods

### Ethical approval

The study has been approved by the Ethics Committee of the Faculty of Medicine Charité Berlin (EA2/077/20; EA4/022/23) and was performed in accordance with the Helsinki Declaration of 1964 as revised in 2013. 

### Inclusion and exclusion criteria

For this retrospective study, patients who underwent segmental resection of the mandible due to malignant or benign tumor or osteonecrosis and received reconstruction with a CAD/CAM SFF and PSI(s) at Charité – Universitätsmedizin Berlin were eligible for inclusion. The inclusion period was between January 2019 and January 2022. Patients who underwent resection of one or both mandibular condyles were excluded, as were patients in whom an intraoperative deviation from the virtual plan was observed.

### Virtual planning 

The CAD/CAM planning procedure was conducted as previously described for FFF [16, 32]. Preoperative computed tomography (CT) or cone beam CT (CBCT) scans of the mandible and thorax were performed to generate 3D datasets for subsequent virtual surgical planning. For patients with malignancies, the treatment protocol was established after staging was completed during an interdisciplinary tumor board conference. For each patient, DICOM data from both donor and recipient sites were uploaded to a cloud-based planning platform (KLS Martin, Tuttlingen, Germany) with a slice thickness of up to 1 mm. Based on radiological and clinical evaluations, resection margins were determined by a senior consultant from the Department of Oral and Maxillofacial Surgery, Charité – Universitätsmedizin Berlin (Germany). Virtual planning encompassed flap reconstruction design and the creation of customized drilling and cutting guides for both the resection and flap donor sites. This planning was performed collaboratively during web-based meetings with engineers from the manufacturer (KLS Martin Group) and senior surgical consultants. The titanium reconstruction plate thickness was specified with a layer thicknesses of 2.0 to 2.5 mm (KLS Martin, Tuttlingen, Germany) [33] and locking screws with individual lengths. Cutting and drilling guides were fabricated using 3D printing technology, while the reconstruction plates were manufactured via a laser-melting process, ensuring precise adaptation to patient-specific anatomical requirements.

### Surgery

In a first step, the resection of the mandible was carried out as virtually planned. After dissection of the cervical vessels in preparation for subsequent anastomosis, patients were positioned on their side (contralateral to the donor site) to harvest the SFF. After guide fixation, a piezoelectric bone-cutting device was used for all osteotomies (Piezosurgery, Mectron, Cologne, Germany). After raising of the flap, the primary wound closure followed, and the patient was placed on the back again. Then, the reconstruction plate was fixated to the flap with locking screws of individual length and reconstruction of the mandible was carried out. Arterial anastomosis was performed using Prolene 8.0 single-button sutures. For venous anastomosis, either Prolene 8.0 sutures or a coupler system was used. Intraoral and extraoral wound closure, anticoagulation with fraxiparine^®^ 0.3 mL 1-0-1, nutrition via a gastric tube and protective tracheostomy was carried out as previously described [16]. After discharge, outpatient controls with clinical examination were performed on a regular basis, including at least one CT or CBCT control, which was retrospectively used to assess the accuracy of the postoperative results.

### 3-Dimensional accuracy

**Fig. 1:**
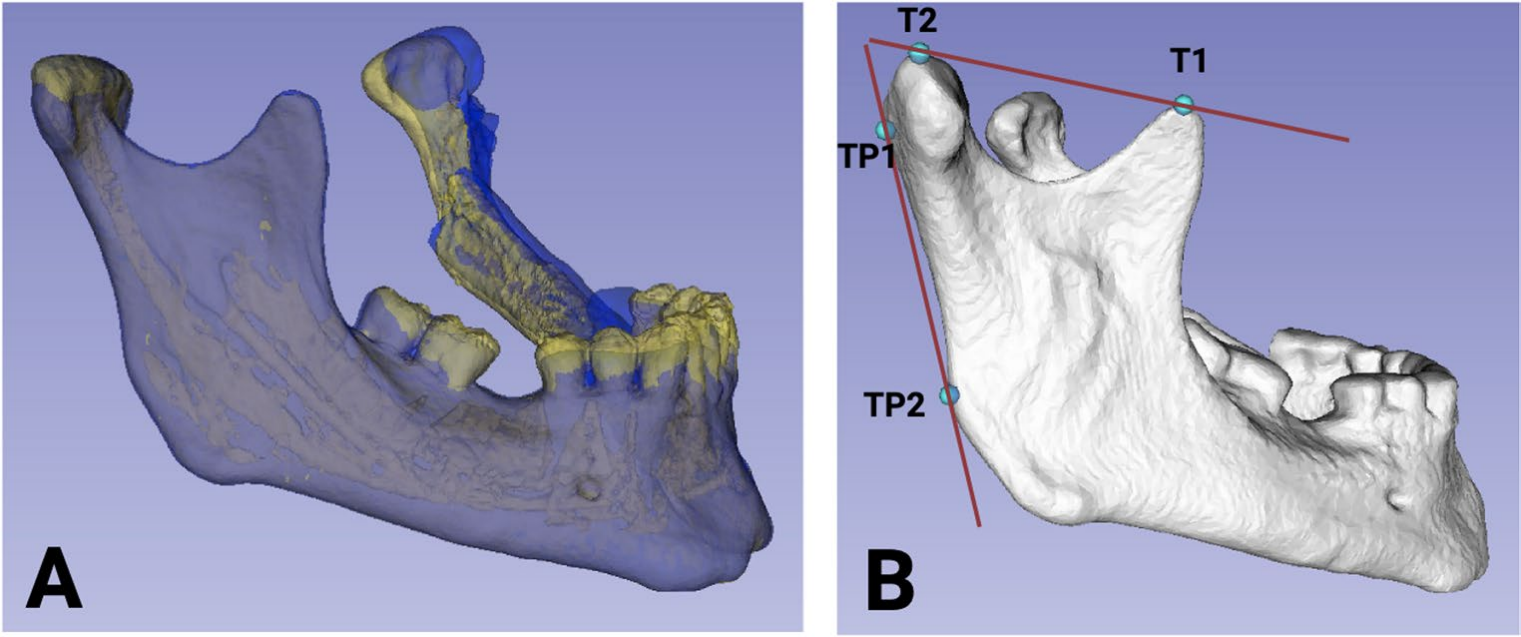
(**A**) Superimposition of the preoperative plan (blue) and the postoperative outcome (yellow). (**B**) A tangent was defined along the cranial and posterior regions, with two contact points in each region (T1 and T2, as well as TP1 and TP2) used for analysis. (Created with Biorender.com)

The accuracy measurements were performed three times by independent investigators with the software 3D slicer (https://download.slicer.org/), a free and open-source platform for analyzing medical image data [34]. To evaluate the concordance between the preoperative plan and the postoperative outcome, an overlay of the respective datasets was performed. Alignment was achieved using the mandibular side with the smaller resection extent to provide a precise basis for comparison. The preoperative plan was represented in the overlay by blue markings, while the postoperative outcome was depicted in yellow (Fig. 1 A). The accuracy of the matching process was thoroughly assessed. A tangent was defined along the cranial and posterior regions (Fig. 1B), with two contact points in each region used for analysis. The posterior tangent established two contact points with the ascending mandibular ramus, whereas the cranial tangent contacted the condylar head and the coronoid process of the mandible. Deviations between the preoperatively planned and postoperatively achieved positions were measured in millimeters to quantify execution precision.

**Fig. 2:**
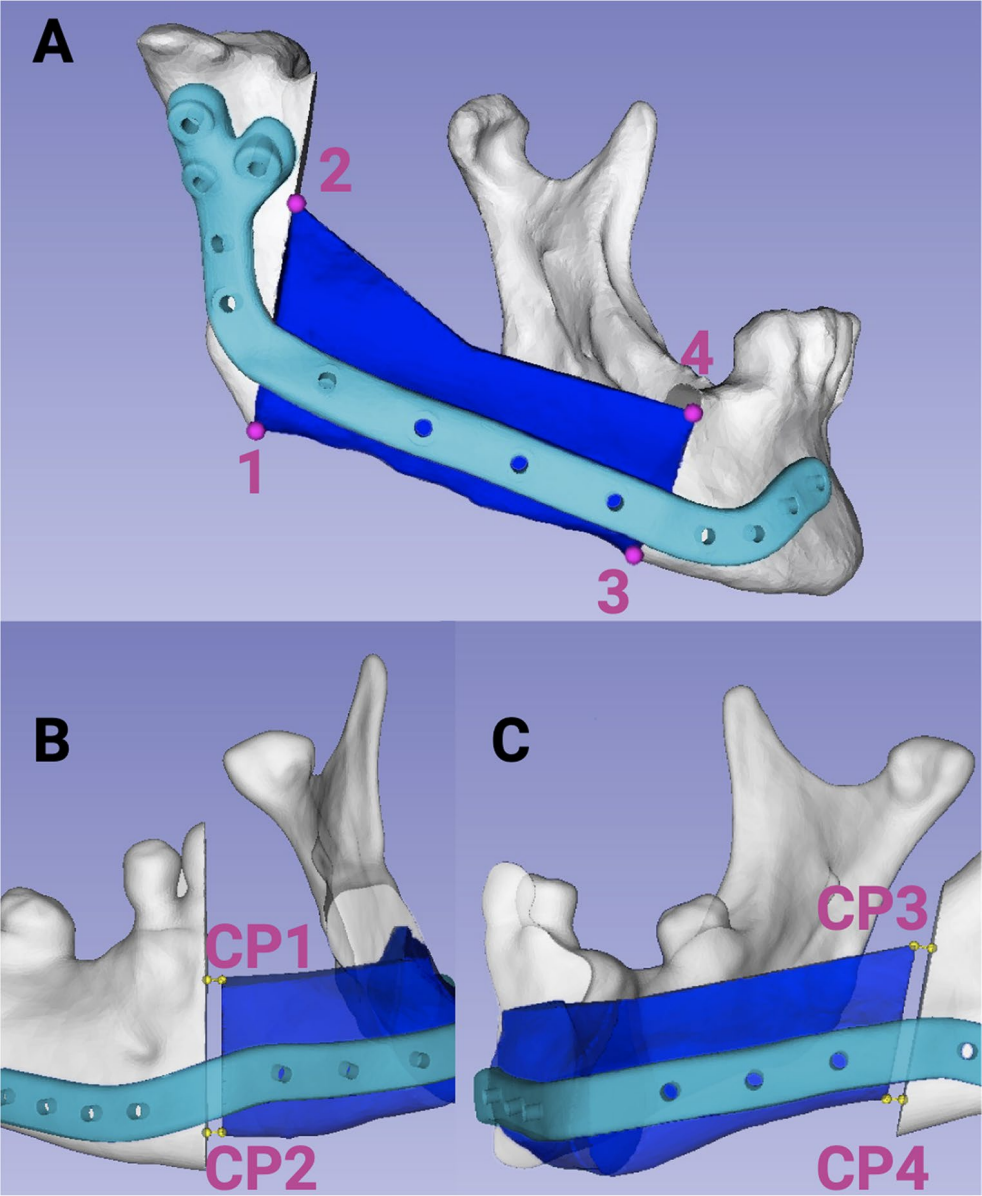
(**A**) Evaluation of the accuracy of segment positioning was conducted by identifying four reference points: two at the posterior border (Points 1 and 2) and two at the anterior border (Points 3 and 4). (**B** and **C**): The accuracy of the gap width between the anterior and posterior segments of the SFF and the mandible was also assessed. Four points were defined at the anterior basal and cranial scapular segment (CP1 and CP 2), as well as the posterior basal and cranial scapular segment (CP3-CP4). From this point, a horizonal perpendicular was designed and the distance to the mandible was measured to receive the segmental gap width. All deviations were recorded in millimeters. (Created with Biorender.com)

To further evaluate segment position accuracy, four specific points were identified: two at the posterior border (Points 1 and 2; Fig. 2A) and two at the anterior border (Points 3 and 4; Fig. 2A) between the scapular and mandibular bone. If no direct contact between the mandible and scapula was observed, the basal and crestal tips at the anterior and posterior scapular borders were used to determine the accuracy of the scapular segment. The accuracy of the segmental gap width was assessed by measuring the gap between the anterior and posterior border of the SFF segment and the mandible as a three-dimensional vector (Fig.2B, C). Generally, no gap between the mandible and the scapular bone was planned (segmental gap 0.0 mm). For multi-segment grafts, only the contact at the mandible–scapula interface was quantified to allow for comparability. To measure the preoperative or postoperative segmental gap, four points were defined at the anterior basal and cranial scapular segment (CP1 and CP 2), as well as the posterior basal and cranial scapular segment (CP3-CP4) (Fig. 2B and C). From this point, a horizonal perpendicular was designed and the distance to the mandible was measured to receive the segmental gap width in mm. This was carried out in the same manner for preoperative and postoperative segmental gaps. Precise measurements of both the planned and postoperative segmental gaps were taken in millimeters to evaluate congruence and deviations. Additionally, condylar head movement was measured in millimeters in three-dimensional space.

### Osseous union and plate related complications

Plate related complications, such as intra- and extraoral plate exposure, plate loosening, and plate fracture were assessed clinically and radiographic (CT, CBCT, orthopantomogram) as described before [35]. Using postoperative 3D-Imaging (CT, CBCT) osseous consolidation was examined at a time windows (after 12–24 months) by two independent observers, as in a previous study [16].

### Statistics

For statistical analysis the data were tested for normal distribution with Shapiro-Wilk-Test. The test results revealed that the data were not normally distributed. Accordingly, the non-parametric Wilcoxon Rank sum test for dependent samples was used and the results were adjusted with holm correction for multiple testing. All statistical analyses were performed with R (Version 2024. 12.0), and the significance level was set at p < 0.05.

## Results

### Patient characteristics

A total of 15 patients met the inclusion criteria (7 women, 8 men, mean age 65.7 ± 10.0 years, range 34–81 years). Reconstruction of the mandible was mostly required due to malignancies (86.7%; *n* = 13), in one patient due to ameloblastoma (6.7%), and in one patient (6.7%) due to osteoradionecrosis of the jaw. The number of used segments varied, with two segments (53.3%; *n* = 8) and 1 segment (40.0%; *n* = 6) occurring most frequently. Three segments were only found in one patient (6.7%). The first adequate radiological follow up was performed 4.4 ± 4.3 months after surgery.

### Accuracy

To assess the accuracy of the matching (planning and result), the two contact points of the posterior (ascending ramus of the mandible) and cranial tangent (condylar head and coronoid process of the mandible) were defined and their discrepancy was determined. The mean distance for T1 was 0.75 ± 0.36 mm; T2 0.85 ± 0.49 mm, TP1 0.94 ± 0.50, and TP2 0.68 ± 0.35 mm (Table 1). Determination of the accuracy of the segments showed notable deviations, especially in the posterior region (basal: 6.78 ± 3.34 mm; crestal: 7.61 ± 3.40 mm) when compared to the anterior region (basal: 3.54 ± 1.52 mm; crestal: 4.49 ± 2.51 mm) (Table 2). Subsequently, the deviation from the planned segmental gap (anteriorly and posteriorly) was measured at four measuring points. Here, for CP1 (0.55 ± 0.85 mm; *p* < 0.01), CP2 (0.87 ± 1.21 mm; *p* < 0.01), CP3 (1.72 ± 2.20 mm; *p* < 0.01) and CP4 (1.01 ± 1.26 mm; *p* < 0.01) the deviation was found to differ significantly from the planned segmental gap (Table 3). Since in all patients both condyles were preserved, accuracy of the condyle positioning of the non-matching side was measured (Table 4). With a mean difference of 8.56 ± 4.31 mm, the condyle position differed significantly from the initial position (*p* < 0.01).

**Table 1:** Matching accuracy. Using a tangent at the cranial (T1 and T2) and posterior region (TP1 and TP2), the matching accuracy was measured with the deviation at each of the four defined points. For each patient, the deviation is reported in mm. In addition, the mean value (Mean) and the standard deviation (SD) are provided for each tangent point

	Tangent point 1 in mm	Tangent point 2 in mm	Tangent point 3 in mm	Tangent point 4 in mm
Pat. No.	T1	T2	TP1	TP2
1	0.38	1.16	0.58	0.62
2	0.59	0.57	0.16	0.28
3	0.68	1.33	0.74	1.06
4	1.23	0.96	0.77	0.22
5	0.63	0.49	0.80	0.78
6	0.51	0.83	0.56	0.19
7	1.37	1.50	1.83	1.06
8	0.47	0.18	1.76	0.80
9	0.86	0.46	1.46	0.94
10	0.23	1.52	1.59	1.29
11	0.61	0.39	0.37	0.18
12	0.26	0.31	0.36	0.36
13	1.07	0.22	0.93	0.61
14	1.37	1.27	1.02	0.86
15	0.92	1.54	1.1	0.93
Mean	**0.75**	**0.85**	**0.94**	**0.68**
SD	0.36	0.49	0.50	0.35

**Table 2:** Deviation of scapular segments. Mean value (Mean) ± standard deviation (SD) in millimeters (mm) of the scapular segments at the four measuring points at the posterior and anterior border (basal and crestal) for each patient. Additionally, the mean and the SD of all patients are given

	Basal posterior (mm)	Crestal posterior (mm)	Basal anterior (mm)	Crestal anterior (mm)
Pat. No.				
1	13.50 ± 0.40	15.69 ± 0.24	1.99 ± 0.08	2.12 ± 0.06
2	4.69 ± 0.30	4.09 ± 0.22	2.94 ± 0.08	7.53 ± 0.11
3	4.49 ± 0.39	6.36 ± 0.25	5.06 ± 0.24	6.21 ± 0.23
4	0.93 ± 0.05	1.85 ± 0.20	3.94 ± 0.26	2.38 ± 0.56
5	9.34 ± 0.31	5.83 ± 0.07	5.83 ± 0.24	4.59 ± 0.13
6	7.67 ± 0.16	8.72 ± 1.69	4.26 ± 0.18	3.86 ± 0.10
7	13.70 ± 0.18	10.16 ± 0.20	4.46 ± 0.17	1.28 ± 0.10
8	6.79 ± 0.30	10.75 ± 0.38	2.35 ± 0.15	6.60 ± 0.24
9	6.81 ± 0.28	2.86 ± 0.18	2.95 ± 0.22	3.20 ± 0.23
10	5.90 ± 0.30	4.71 ± 0.04	1.66 ± 0.13	2.05 ± 0.08
11	8.11 ± 0.18	8.15 ± 0.26	3.46 ± 0.12	3.14 ± 0.12
12	5.85 ± 0.35	7.80 ± 0.18	5.77 ± 0.19	4.55 ± 0.19
13	2.99 ± 0.26	8.75 ± 0.16	1.04 ± 0.08	11.22 ± 0.07
14	4.46 ± 0.11	10.27 ± 0.11	1.92 ± 0.26	3.29 ± 0.07
15	6.51 ± 0.29	8.15 ± 0.11	5.38 ± 0.11	5.29 ± 0.04
Mean	**6.78**	**7.61**	**3.54**	**4.49**
SD	3.34	3.40	1.52	2.51

**Table 3:** Segmental gap width. All mean values ± standard deviation for the planned (preoperative = preop.) and postoperative (= postop.) segmental gap width as well as the deviation of the segmental gap width in mm for each patient are listed. Additionally, the mean value (Mean) and the standard deviation (SD) for all 15 patients are given

	Anterior Crestal Gap Width			Anterior Basal Gap Width			Posterior Crestal Gap Width			Posterior Basal Gap Width		
	Preop.	Postop.	Deviation	Preop.	Postop.	Deviation	Preop.	Postop.	Deviation	Preop.	Postop.	Deviation
Pat. No.	CP1			CP2			CP3			CP4		
1	0.00 ± 0.00	0.79 ± 0.17	0.79 ± 0.17	0.00 ± 0.00	1.78 ± 0.15	1.78 ± 0.15	0.00 ± 0.00	1.49 ± 0.11	1.49 ± 0.11	0.00 ± 0.00	0.00 ± 0.00	0.00 ± 0.00
2	0.00 ± 0.00	0.00 ± 0.00	0.00 ± 0.00	0.00 ± 0.00	0.00 ± 0.00	0.00 ± 0.00	0.00 ± 0.00	2.58 ± 0.24	2.58 ± 0.24	0.00 ± 0.00	2.56 ± 0.49	2.56 ± 0.49
3	0.00 ± 0.00	0.00 ± 0.00	0.00 ± 0.00	1.33 ± 0.11	0.00 ± 0.00	1.33 ± 0.11	0.00 ± 0.00	0.00 ± 0.00	0.00 ± 0.00	0.00 ± 0.00	2.60 ± 0.36	2.60 ± 0.36
4	0.00 ± 0.00	0.00 ± 0.00	0.00 ± 0.00	0.00 ± 0.00	0.00 ± 0.00	0.00 ± 0.00	0.00 ± 0.00	0.00 ± 0.00	0.00 ± 0.00	0.00 ± 0.00	0.00 ± 0.00	0.00 ± 0.00
5	0.00 ± 0.00	0.00 ± 0.00	0.00 ± 0.00	0.00 ± 0.00	0.00 ± 0.00	0.00 ± 0.00	0.00 ± 0.00	2.29 ± 0.11	2.29 ± 0.11	0.00 ± 0.00	0.00 ± 0.00	0.00 ± 0.00
6	0.00 ± 0.00	1.28 ± 0.29	1.28 ± 0.29	0.00 ± 0.00	1.26 ± 0.07	1.26 ± 0.07	0.00 ± 0.00	2.52 ± 0.02	2.52 ± 0.02	0.00 ± 0.00	0.00 ± 0.00	0.00 ± 0.00
7	0.00 ± 0.00	0.00 ± 0.00	0.00 ± 0.00	0.00 ± 0.00	0.44 ± 0.38	0.44 ± 0.38	0.00 ± 0.00	0.85 ± 0.10	0.85 ± 0.10	0.00 ± 0.00	3.92 ± 0.10	3.92 ± 0.10
8	0.00 ± 0.00	0.00 ± 0.00	0.00 ± 0.00	0.00 ± 0.00	0.00 ± 0.00	0.00 ± 0.00	0.00 ± 0.00	6.83 ± 0.15	6.83 ± 0.15	0.00 ± 0.00	0.00 ± 0.00	0.00 ± 0.00
9	0.00 ± 0.00	0.93 ± 0.10	0.93 ± 0.10	0.00 ± 0.00	0.00 ± 0.00	0.00 ± 0.00	0.00 ± 0.00	1.97 ± 0.04	1.97 ± 0.04	0.00 ± 0.00	1.96 ± 0.03	1.96 ± 0.03
10	0.00 ± 0.00	0.00 ± 0.00	0.00 ± 0.00	0.00 ± 0.00	0.00 ± 0.00	0.00 ± 0.00	0.00 ± 0.00	0.00 ± 0.00	0.00 ± 0.00	0.00 ± 0.00	0.00 ± 0.00	0.00 ± 0.00
11	0.00 ± 0.00	0.00 ± 0.00	0.00 ± 0.00	0.00 ± 0.00	0.00 ± 0.00	0.00 ± 0.00	0.00 ± 0.00	0.00 ± 0.00	0.00 ± 0.00	0.00 ± 0.00	0.00 ± 0.00	0.00 ± 0.00
12	2.02 ± 0.04	0.93 ± 0.10	1.09 ± 0.14	2.06 ± 0.08	0.45 ± 0.11	1.62 ± 0.18	2.17 ± 0.06	2.59 ± 0.29	0.42 ± 0.28	2.10 ± 0.07	0.00 ± 0.00	2.10 ± 0.07
13	0.00 ± 0.00	0.00 ± 0.00	0.00 ± 0.00	0.00 ± 0.00	0.90 ± 0.01	0.90 ± 0.01	4.04 ± 0.06	10.73 ± 0.20	6.69 ± 0.26	0.00 ± 0.00	0.00 ± 0.00	0.00 ± 0.00
14	0.00 ± 0.00	3.13 ± 0.08	3.13 ± 0.08	0.00 ± 0.00	1.06 ± 0.06	1.06 ± 0.06	0.00 ± 0.00	0.18 ± 0.03	0.18 ± 0.03	0.00 ± 0.00	0.96 ± 0.03	0.96 ± 0.03
15	0.00 ± 0.00	1.01 ± 0.07	1.01 ± 0.07	0.00 ± 0.00	4.68 ± 0.16	4.68 ± 0.16	0.00 ± 0.00	0.00 ± 0.00	0.00 ± 0.00	0.00 ± 0.00	1.10 ± 0.06	1.10 ± 0.06
Mean	0.13	0.54	**0.55**	0.23	0.70	**0.87**	0.41	2.13	**1.72**	0.14	0.87	**1.01**
SD	0.50	0.84	0.85	0.59	1.20	1.21	1.11	2.89	2.20	0.52	1.25	1.26
*p*-value	-	-	**< 0.01**	-	-	**< 0.01**	-	-	**< 0.01**	-	-	**< 0.01**

**Table 4:** Number of segments and mean movement of the condyle on the non-matching side in mm. Additionally, the number of segments is given for each patient, and the mean value (Mean) and the standard deviation (SD) of all 15 patients are listed

	Number of segments	Deviation in mm non-matching Condyle
Pat. No.		
1	1	8.52
2	2	3.32
3	3	6.88
4	1	3.50
5	1	8.04
6	1	15.18
7	2	19.57
8	1	3.78
9	2	8.79
10	2	8.94
11	2	8.59
12	2	3.94
13	2	8.14
14	1	9.18
15	2	11.96
Mean	**1.67**	**8.56**
SD	0.60	4.31
*p*-value	-	< 0.01

Furthermore, to determine interrater-reliability, intraclass-correlation was tested and revealed an intraclass correlation coefficient (ICC) of 0.99 for anterior crestal gap width, 0.89 for anterior basal gap width, 0.99 for posterior crestal gap width and 0.99 for posterior basal gap width.

### Osseous union and plate related complications

Among the 15 patients included in this study, 3 patients (20%) experienced plate-related complications, all of which involved intraoral plate exposure. None of these patients had received adjuvant radiotherapy. Intraoral plate exposure occurred at 19, 193, and 648 days postoperatively, respectively. The corresponding mean deviations from the planned segmental gap in these patients were as follows: CP1: 0.31 ± 0.53 mm, CP2: 0.44 ± 0.76 mm, CP3: 1.42 ± 1.24 mm, and CP4: 1.52 ± 1.35 mm. Segment positioning accuracy in this subgroup showed mean deviations of 6.88 ± 2.43 mm (basal) and 5.02 ± 1.89 mm (crestal) in the posterior region, and 4.61 ± 1.49 mm (basal) and 4.67 ± 1.51 mm (crestal) in the anterior region. The mean deviation in condylar position was 7.90 ± 0.96 mm. No extraoral plate exposure, plate fracture, or plate loosening was observed in any case.

Osseous union data were available for 10 patients (67%) after 12–24 months of follow-up. In detail, two patients were lost to follow up, one patient died, one patient experienced flap loss prior to the segment-union analysis imaging period (6–24 months postoperatively) and one patient first took follow-up consultations after > 24 months (thereby exceeding the 6–24 months analysis range). Regarding sex (female: 40% in non-imaging vs. 50% in imaging group), postoperative radiotherapy (20% in non-imaging vs. 20% in imaging group) and age (64.4 ± 17.0 years in non-imaging vs. 67.2 ± 5.7 in imaging group) no relevant differences were found. Patients in the non-imaging group received one- (60%) or two-segmental flaps (40%), while patients in the imaging group received one- (30%), two- (60%) or three-segmental flaps (10%). Patients in the non-imaging group underwent anamnestic radiotherapy more regularly (60% vs. 30%), while patients in the imaging group were abusing nicotine more regularly (30% vs. 0%). Malignant diseases were the main indication in both groups (80% non-imaging group vs. 90% imaging group), with single cases of benign disease (non-imaging group) and osteoradionecrosis (imaging group). Among the 10 patients, 3 patients (30%) showed non-union between the mandible and the SFF, one of whom had received adjuvant radiotherapy. No osseous non-union between flap segments was recorded. In patients with osseous non-union, the mean postoperative segmental gap was 0.34 ± 0.58 mm (CP1), 1.71 ± 2.58 mm (CP2), 1.05 ± 1.16 mm (CP3), and 2.07 ± 1.97 mm (CP4). In patients with non-union, the segment positioning accuracy was markedly reduced, with mean deviations in the posterior region of 9.85 ± 3.62 mm (basal) and 8.05 ± 2.17 mm (crestal), and in the anterior region of 5.22 ± 0.70 mm (basal) and 3.72 ± 2.14 mm (crestal). The mean deviation of the condylar position in these cases was 13.19 ± 5.86 mm.

## Discussion

Today, CAD/CAM assisted reconstruction of the head and neck region, especially regarding osseous reconstruction of critical-sized bone defects, has been successfully implemented in the routine workflow of most departments [36, 37]. Previous studies already highlighted the high accuracy, as well as the high esthetic and functional results of this workflow [25, 27, 28, 30, 31]. Regarding CAD/CAM assisted osseous reconstruction of the mandible, most studies focus on the “gold standard”, the FFF [27–29], or the DCIA flap [30]. Unfortunately, these flaps are not always suitable for all patients, e.g. if peripheral vascular disease, or mandibular defects associated with complex soft-tissue requirements are present. In such cases, the SFF might offer a valid option for reconstruction, but studies on the accuracy of SFF in CAD/CAM assisted surgery are still rare, since only a limited number of articles on mandibular reconstruction with CAD/CAM assisted SFF exist [26, 38].

In our study, the accuracy of segment position differed between the anterior and the posterior aspect. Anteriorly, the position differed between 3.54 (basal mean) and 4.49 mm (crestal mean), and the posterior measuring points revealed a clearly higher deviation (basal: 6.78 mm; crestal: 7.61 mm). Comparing the accuracy of CAD/CAM assisted mandibular reconstruction, various methods have been described [16, 39–41]. Regarding mandibular reconstruction, a systematic review of van Baar et al. revealed several influencing factors for deviations of the postoperative result when compared to the preoperative planning [26]. Including 42 studies with 413 mandibular reconstruction, the conclusion was drawn that inaccuracies are introduced at various stages, such as image acquisition, segmentation, 3D printing, surgery, and evaluation of postoperative results [26, 42, 43]. With deviations between 0 and 12.5 mm [26], the observed deviations in the presented study regarding the position of the scapula segments seems to be comprehensible. Nevertheless, these deviations must be set in relation to the used flap. Thus, most studies mainly focus on FFF or DCIA; correspondingly the 42 studies with 413 mandibular reconstructions included only one SFF [26]. Since the SFF usually have a relatively large soft tissue cuff around the bone, the fitting of the cutting template and therefore the subsequent reconstruction is directly affected. This might contribute to the already previously described deviations of the post-operative results from the pre-operative planning [26]. Nonetheless, this also harbors opportunities, such as “open” osteotomies to create an open wedge at the labial surface, while leaving the lingual cortex of the bone and periosteum intact [44].

Despite the relatively low number of dental implant insertion into scapula bone [44, 45], dental implant insertion is of particular importance during the patient’s prosthodontic rehabilitation [46, 47], and therefore this aspect needs to be involved in CAD/CAM planning procedure/VSP. This is underlined by several studies which report on the possibility of dental implant placement in patients after reconstruction with a scapular/parascapular flap [48–50].

In our study, the segments showed notable deviations, especially in the posterior region when compared to the anterior region. Nevertheless, especially in the anterior region, these deviations should allow successful dental implant placement if permitted by the bone supply in these regions. Another point of interest in osseous reconstruction of the mandible is the ossification between the transplant and the recipient site. For STFF, complete or partial osseous union between the mandible and the scapular graft is reported to be relatively high with approximately 92% [44]. Other studies investigating osseous union in STFF have reported lower union rates, ranging from 74.4% (proximal osteotomy) to 82.6% (distal osteotomy); in another study 11 non-unions were reported among 80 patients [51, 52]. In the present study, osseous union was assessed after 12–24 months, revealing osseous union in 7 out of 10 patients, which falls within the range reported in the literature comparing free bone flaps in reconstruction of the jaw [33, 53]. Regarding osseous union between the mandible and the scapular graft in particular, Barton et al. reported complete or partial union at the proximal site in 20 of 25 patients (80%) and at the distal site in 21 of 25 patients (84%) [44], which is also consistent with our findings. In addition, all included patients were evaluated for plate-related complications. Here, intraoral plate exposure was observed in three cases, while no other complications such as plate fracture or loosening were identified. The incidence of intraoral plate exposure in this cohort is consistent with findings from previous studies, including those involving FFF reconstructions with reconstruction plates (PSI) [54] and osseous free flaps in general [53]. Regarding segment positioning, patients with plate exposure showed mean deviations of 6.88 ± 2.43 mm (basal) and 5.02 ± 1.89 mm (crestal) in the posterior region, and 4.61 ± 1.49 mm (basal) and 4.67 ± 1.51 mm (crestal) in the anterior region, with only three measurement points slightly exceeding the overall mean. Accordingly, a greater deviation in segment positioning may be associated with an increased risk of plate exposure. However, due to the small sample size, only a descriptive assessment was feasible. In addition to the osseous (non-) union and plate related complications, we evaluated the accuracy of virtual planning transmission and the actual segmental gap in CAD/CAM-assisted surgery using PSI. Most studies on the width of the segmental gap focus on the FFF, as it remains the “gold standard” for mandibular reconstruction. Here, Hashemi et al. reported that healing of the neomandible appeared to be improved with gap sizes < 2 mm and suggest a higher risk of non-union if the osteotomy gap is larger than 2.55 mm [55]. Therefore, the importance of the transmission of the planned segmental gap becomes obvious. The mean deviation at the anterior gap region was 0.55 mm, and 0.87 mm, respectively 1.01 mm, and 1.72 mm posteriorly. This indicates that even despite the relatively large soft tissue cuff around the scapular bone and the resulting possible inaccuracies regarding the fitting of CAD/CAM cutting guides and the PSI, a segmental gap width enabling osseous union can be reached.

For accuracy measurements, Van Baar et al. identified the condyle (54%) as the most common landmark [26], which was also considered in this study. In a study on mandibular reconstruction with flaps from the scapular systems, Harada et al. reported a low number of mandibular condyle dislocation (around 2%) [45]. Despite no condyle dislocation was found in our study, we identified significant changes in the contralateral condyle position with movements of up to a maximum of 19.57 mm (mean 8.56 ± 4.31 mm). With respect to the number of segments, no association between the number of segments and deviation of the non-matching condyle was observed. For example, in one patient reconstructed with a single segment, a mean deviation of 8.52 mm was measured, whereas in a patient reconstructed with three segments, the mean deviation was 6.88 mm. Since only one point of the condyle was determined regarding the condyle position, this could also lead to the relatively high deviations due to inaccuracies. In another study for example, deviations were also found between different investigators [56], indicating how difficult a reproducible and uniform determination of accuracy is. Nevertheless, the values in our study for the intraclass correlation of preoperative and postoperative deviations showed strong results, i.e. high interrater reliability.

Moreover, it was found that comparing the accuracy in mandibular reconstruction revealed higher deviations in the condyle area when compared to other regions of the mandible [56]. During reconstruction, CAD/CAM reconstruction plates (PSI) were used to fixate the SFF and connect the two remaining parts of the mandible-and since small deviations at the time of template application (matching mandible side) and pre-drilling can cause a relatively large deviation on the contralateral side, this must also be considered. In patients with osseous non-union, the measured deviation of the condyle position was 13.19 ± 5.86 mm, which was greater compared to the remaining patients with osseous union. A similar pattern was observed for segment positioning accuracy: in non-union cases, deviations were particularly pronounced in the posterior region, which may adversely affect osseous consolidation. Whether, and to what extent, the deviation of the condylar position and posterior segmental width plays a role should be further investigated in prospective studies.

The limitations of this study certainly include the small number of included patients and the lack of a control group. These limitations might be overcome by the fact that (to the best of the authors’ knowledge) this study is the first study on the accuracy of SFF in CAD/CAM reconstruction of the mandible. The findings of this study indicate that SFF with patient-specific 3D-printed reconstruction plates can be conducted with an acceptable accuracy for clinical application, particularly in the anterior region, while relevant deviations in the posterior and condylar regions remain and warrant further optimization.

## Data Availability

All data supporting the findings of this study are available within the article. Additional de-identified data may be made available by the corresponding author upon reasonable request.
